# Efficacy of a transdiagnostic guided internet-delivered intervention for emotional, trauma and stress-related disorders in Mexican population: study protocol for a randomized controlled trial

**DOI:** 10.1186/s12888-022-04132-6

**Published:** 2022-08-09

**Authors:** Anabel de la Rosa-Gómez, Lorena A. Flores-Plata, Esteban E. Esquivel-Santoveña, Carolina Santillán Torres Torija, Raquel García-Flores, Alejandro Dominguez-Rodriguez, Paulina Arenas-Landgrave, Rosa O. Castellanos-Vargas, Enrique Berra-Ruiz, Rocío Silvestre-Ramírez, Germán Alejandro Miranda-Díaz, Dulce M. Díaz-Sosa, Alejandrina Hernández-Posadas, Alicia I. Flores-Elvira, Pablo D. Valencia, Mario F. Vázquez-Sánchez

**Affiliations:** 1 Faculty of Higher Studies Iztacala, National Autonomous University of Mexico, State of Mexico, Mexico; 2 Department of Social Sciences, Autonomous University of Juarez, Ciudad Juárez, Mexico; 3 Department of Psychology, Technological Institute of Sonora, Sonora, Mexico; 4 Health Sciences Area, Valencian International University, Valencia, Spain; 5 Faculty of Psychology, National Autonomous University of Mexico, Mexico City, Mexico; 6 Health Sciences Department, Autonomous University of Ciudad Juarez, Ciudad Juárez, Mexico; 7 Faculty of Health Sciences, Autonomous University of Baja California, Tijuana Baja California, Mexico; 8 Health of Tlaxcala, State Coordination of Mental Health, Tlaxcala, Mexico

**Keywords:** Emotional disorders, Trauma disorders, Transdiagnostic, Internet-based intervention, Telepsychology

## Abstract

**Background:**

Emotional and stress-related disorders show high incidence, prevalence, morbidity, and comorbidity rates in Mexico. In recent decades, research findings indicate that cognitive behavioral interventions, from a disorder-specific perspective, are the effective front-line treatment for anxiety and depression care. However, these treatments are not often used. Reasons include limited access and low availability to effective interventions and comorbidity between mental disorders. Emotional deregulation of negative affectivity has been found to be a mediating factor in addressing emotional disorders from a transdiagnostic perspective, aimed at two or more specific disorders. In addition, technological advancement has created alternatives for psychological assistance, highlighting the possibilities offered by technologies since Internet-supported intervention programs have been empirically tested for effectiveness, efficiency and efficacy and can be key to ensuring access to those who are inaccessible. The aim of the study is to evaluate the efficacy, moderators of clinical change and acceptability of a transdiagnostic guided Internet-delivered intervention versus a transdiagnostic self-guided Internet-delivered intervention for emotional, trauma and stress-related disorders, and waiting list in community sample.

**Methods:**

A three-armed, parallel group, superiority randomized controlled clinical trial with repeated measurements at four times: pretest, posttest, follow-up at 3, 6 and 12 months. Outcomes assessor, participant, care provider and investigator will be blinded. Participants aged 18 to 70 years will be randomly allocated 1:1:1 to one of three study arms: a) Transdiagnostic guided internet-delivered intervention with synchronous assistance, b) Transdiagnostic self-guided internet-delivered intervention, c) Waiting list group. Based on sample size estimation, a minimum of 207 participants (69 in each intervention group) will be included.

**Discussion:**

The study could contribute to improving the efficacy of transdiagnostic internet-delivered interventions to promote the dissemination of evidence-based treatments and eventually, to decrease the high prevalence of emotional and trauma-related disorders in the Mexican population.

**Trial registration:**

ClinicalTrial.gov: NCT05225701. Registered February 4, 2022.

## Background

Globally, mental disorders are a serious public health problem with a high social cost that affects people regardless of age, sex, socioeconomic status, or culture [1]. In particular, Emotional Disorders (ED), defined as anxiety and mood disorders (unipolar depression), have been identified as the main causes of psychological disability [2], due to their high prevalence, morbidity and comorbidity. In this regard, the Pan American Health Organization (PAHO) revealed high prevalence estimates for anxiety disorders (28.8%) and mood disorders (20.8%), with comorbidity rates ranging from 40 to 80% [3]. In Mexico, the last National Survey of Psychiatric Epidemiology, in 2003 reported that among the most common disorders of the population are those related to anxiety (14.3%) [4]; while depression accounts for 4.3% of the overall burden of mental disorders [3]. Thus, the impact on the health of the general population, and in particular the Mexican population, lies not only in statistics, but also in the emotional and economic consequences when considering the detrimental effects in the functioning of the person within the family, social and labor spheres, limiting possibilities for personal development and quality of life.

Individuals suffering from depression are more likely to develop comorbidity with other mental disorders. An estimated 53% have concurrent symptomatology with some anxiety disorder [5]. Stress and traumatic events are among the most relevant causes, between 60 and 80%, contributing to the etiology of EDs. Depression and anxiety have also been deemed to create a condition of greater disability by increasing the severity and chronicity rate of psychological discomfort and is associated with increased mortality in medical conditions such as heart disease, chronic-degenerative or psychiatric conditions [6]. In Mexico, one in four people between the age of 18 and 65 have experienced at some point in their lives an ED, but only one in five of those with it receive treatment and the time to receive care in a health center ranges from four to 20 years depending on the reason for consultation [6].

In recent decades, cognitive behavioral interventions (e.g., cognitive restructuring, exposure therapy, anxiety management techniques, cognitive therapy), from a disorder-specific perspective, constitute an effective and first-line treatment for anxiety and depression care [7, 8]. However, they are not often used [9] because of the following reasons: limited access and low availability to effective interventions, a minority of people actively seek psychological care because of their own distress condition, fearing social stigma, geographical barriers that separate them from health centers, time availability, preference for other treatment or self-help, high treatment costs, which makes it inaccessible and unaffordable to both, the user and the public health system [6]. It has also been stated that the comorbidity between mental disorders as well as the gap between research findings and clinical practice could influence the poor dissemination of effective treatments and contribute to a lack of up-to-date professionals providing relevant interventions [10]. This has motivated studies aimed at knowing the moderating, mediating variables and psychological mechanisms that improve the process of clinical change [11]. In addition, the need to implement innovative solutions that contribute to the dissemination of effective treatments for the care of EDs has been raised [8]. In particular, emotional deregulation of negative affection is a factor of interest in research that is providing relevant data for better understanding and approaching EDs from a transdiagnostic perspective, a term coined from a dimensional conception to designate effective treatments targeting two or more specific disorders [12].

In this regard, Barlow returns to the tripartite theory of emotion [13] and proposes a unified transdiagnostic behavioral cognitive protocol for the treatment of EDs with an emphasis on emotional regulation [14]. The Unified Protocol for the Treatment of Emotional Disorders [UP] addresses four main components to decrease emotional dysregulation: emotional avoidance, promotion of cognitive flexibility, exposure to avoided situations and sensations, and emotional awareness focused on the present. UP has shown its effectiveness not only in achieving the decrease in target symptomatology, but also increases attendance at therapeutic sessions compared to conventional psychological interventions [15]. In addition, some meta-analyses have been performed which reveal the effectiveness of transdiagnostic protocols for anxiety disorders [16], and for anxiety and/or depression [17].

In recent years there has been an increase in initiatives aimed at the promotion and intervention in mental health mediated by technology that has favored access and dissemination of effective interventions regardless of distance, physical and social barriers [18]. Currently, internet- delivered psychological interventions can reduce the time of contact between patient and therapist and reaching people who would not otherwise receive treatment [19]. Evidence suggests that Internet-based treatments are effective for the treatment of depression, anxiety, substance abuse and eating disorders [20]. In addition, meta-analysis data reveal that these interventions are as effective as face-to-face treatments [21]. Internet-delivered interventions can be classified into: treatments administered by the therapist, treatments with minimum assistance from the therapist, and fully self-applied treatments [22].

In this sense, there is evidence of effective transdiagnostic internet-delivered interventions focusing on the emotion enhance the scope and impact of psychological treatment programs for emotional disorders [5, 23, 24]. However, there is incipient research into the efficiency of transdiagnostic Cognitive Behavioral Treatments (CBT) via the Internet for Trauma and Stress-related Disorders. Some studies have documented the effectiveness of computer-based interventions showing significant changes in anxiety reduction, presenting moderate effect sizes (*g* = 0.78) and large effect sizes in cases of depression (*g* = 0.84) [7, 25, 26]. Other studies that have investigated the efficacy of transdiagnostic CBT provided over the Internet in cases with concurrent symptomatology of anxiety and depression; have found moderate to large effects for both depression and anxiety *(anxiety: g* = 0.82, 95% CI: 0.58 to 1.05; depression: *g* = 0.79; 95% CI: 0.59–1.00) compared to control groups that included waiting list. However, there are few controlled clinical studies investigating the effects of transdiagnostic treatment via the Internet for ED adapted to the Mexican context and culture.

Evidence of the effectiveness of preventive and/or remedial interventions over the Internet in the context of public health for the reduction of the incidence of depressive and anxious symptomatology is still low; the latest systematic review identified seven uncontrolled clinical studies that evaluated the effect of Internet-mediated interventions with positive results [11]. However, only one clinical study aimed at preventing general anxiety was found, and it did not produce significant results [27], while three studies managed to reduce the incidence of depression [28, 29]. In the study conducted by Dear et al., the authors reported that the self-applied Internet-guided intervention decreased depressive symptomatology in a group of adults and managed to reduce the risk of chronic depression by 39% per follow-up year [29]. Recent research on the effectiveness of self-applied interventions via the Internet with and without the support of a trained psychologist has indicated that fully self-applied technology-mediated treatments show fewer rates of improvement compared to those who did have synchronous support with a therapist [30]. Other studies that have researched the influence of support or guidance during self-applied Internet-based interventions have reported that participants who received weekly support significantly improved in reducing depression compared to a waiting list group, while participants who did not receive support by a psychotherapist did not show significant improvement [31]. However, some authors propose that the differences in results obtained between guided and non-guided interventions are small or non-existent [32]. This aspect is relevant for assessing the efficiency of interventions provided with technology, as they could benefit more people who need it. In general, research findings available in the literature are promising; however, it is also important to note that most studies have been conducted in the Anglo-Saxon or Spanish populations. Hence it becomes necessary to know the results in controlled studies in the Mexican context and culture and with larger samples to reach stronger conclusions regarding the effectiveness and efficiency of transdiagnostic Internet-delivered interventions for the care of EDs.

In Mexico, research in this area is incipient, emphasizing the need to go beyond traditional face-to-face interventions and to design new intervention strategies. In this regard, the possibilities offered by technologies are highlighted since Internet-supported intervention programs have been empirically tested to achieve effectiveness and efficiency/cost–benefit and can be key to ensuring access to those who are inaccessible.

Finally, most of the research that has documented efficacy of transdiagnostic treatments have excluded PTSD from the research, a disorder which continues to be in Mexico more and more prevalent, related to migration, earthquakes, and violence [33]. Transdiagnostic interventions have been used on anxiety and now stress and trauma disorders correspond to a new DSM5 category, and as Gutber et al. [34] underlies, there is a need for trials so we can understand how the transdiagnostic model works with specific symptoms related to PTSD.

### Aims

The primary aim of the study is to evaluate the efficacy and acceptability of a transdiagnostic guided Internet-delivered intervention versus a transdiagnostic self-guided Internet-delivered intervention for emotional, trauma and stress-related disorders, and a waiting list in a Mexican community sample. The second aim is to examine the moderator of clinical change, particularly emotional regulation, in the transdiagnostic Internet-delivered intervention for emotional, trauma and stress-related disorders.

### Hypothesis

- The transdiagnostic guided Internet-delivered intervention will show statistical greater gains in reducing symptoms of anxiety/depression/acute or post-traumatic stress and a more clinically significant improvement compared to the transdiagnostic self-guided Internet-delivered intervention and waiting list groups.
- The transdiagnostic self-guided Internet-delivered intervention will reduce symptoms of anxiety/depression/acute or post-traumatic stress compared to a waiting list group.
- A higher acceptance/satisfaction rate reported by participants in transdiagnostic guided Internet-delivered intervention will be found compared to the transdiagnostic self-guided Internet-delivered intervention.
- Emotional Regulation will be a moderating variable for clinical change.
- Changes will be maintained three, six and 12 months after the end of the intervention program with and without synchronous psychological support.

## Methods

### Study design

**Fig. 1:**
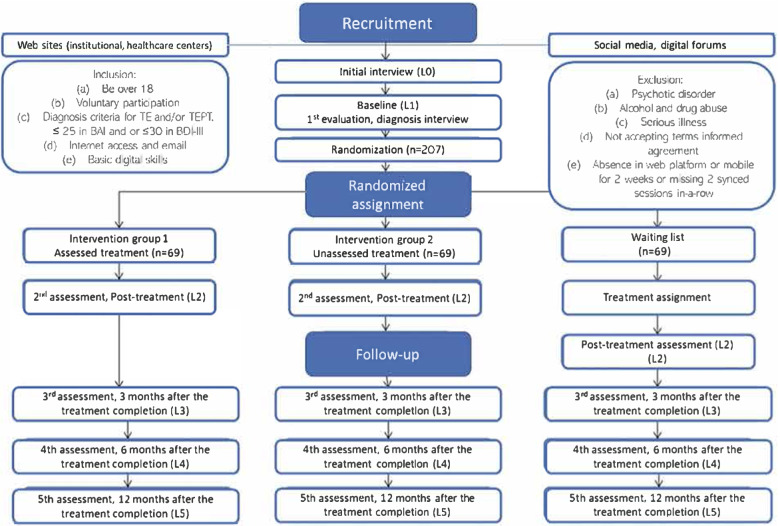
Shows the flow chart of the study design

A three-armed, parallel group, superiority randomized controlled clinical trial with repeated measurements at four times: pretest, posttest, follow-up at three, six and 12 months. Outcomes assessor, participant, care provider and investigator will be blinded. Participants aged 18 to 70 years will be randomly allocated 1:1:1 to one of three study arms: a) Transdiagnostic guided Internet-delivered intervention with synchronous assistance, b) Transdiagnostic self-guided Internet-delivered intervention, c) Waiting list group. This study will follow the CONSORT statement [35] and the SPIRIT guidelines [36]. The study’s trial registration number is *ClinicalTrials.gov* NCT05225701. Figure 1 shows the flow chart of the study design.

### Participants

Sampling: non probabilistic, convenience sampling will be performed. Participants aged 18 to 70 years.

### Eligibility criteria

*Inclusion criteria:* a) be of legal age; b) voluntarily participate in the study; c) meet diagnostic criteria for emotional disorders (anxiety/depression) and trauma, stress-related disorders in accordance with the International Neuropsychiatric Interview- Mini, version 5.0 [37], and show a score ≤ 25 in Beck’s Anxiety Depression Inventory [38] and/or ≤ 30 in the Beck-BDI-II Depression Inventory [39]; d) have access to computer equipment with an Internet connection; e) have a valid email address; f) have basic digital skills in the use of an operating system and Internet browsing.

*Exclusion criteria*: a) psychotic disorder; b) alcohol and drug abuse; c) medical condition whose severity or characteristics prevent the intervention; d) be receiving psychological and/or pharmacological treatment during the study.

*Elimination criteria:* a) not accepting the conditions of informed consent and b) absence on web or mobile platforms for more than 15 days or having missed two consecutive sessions of synchronous treatment sessions.

### Recruitment procedure

Recruitment of participants will be carried out through advertisements in digital media (institutional website, thematic forums), as well as through dissemination on social networks. The Transdiagnostic Internet-delivered intervention program will be aimed at adults who will be able to connect via the Internet from anywhere in Mexico. Potential participants will be contacted via telephone calls or emails. The research coordinator will provide the participants with the information about the nature of the study and clarify doubts. Participants will be asked for their consent to participate. Independent evaluators will determine the suitability of participants to be included in the study based on the initial synchronous interview (via videoconference or telephone) and the self-reports related to the selection criteria that will be available in digital format on the web platform.

### Ethics approval and consent to participate

The project is supported by the Ethics Committee of the Faculty of Higher Studies Iztacala at National Autonomous University of Mexico (*CE/FESI/082020/1363*). The development of the study will strictly adhere to the guidelines expressed in the Mexican Psychologist’s Code of Ethics [40] and the ethical standards that apply to traditional clinical practice and recommendations for online psychotherapy will be observed [41]. The therapist will protect the patient’s confidentiality and interaction records during the therapeutic process. All participants must sign an informed consent form and the rights to the confidentiality and privacy of personal data will be respected. The participant’s personal data will be encrypted through a standard algorithm and accessed only by the principal investigator.

### Randomization and blinding

Participants who meet the inclusion criteria for the study will be randomly assigned to one of the study conditions. Randomization will be performed by an independent researcher unaware of the study characteristics through online open software [42] in a 1:1:1 ratio per block saturation of 12 per condition. The coordinator will inform the patient of their participation in the study and, depending on the characteristics of the study design condition, the user will be assigned to the self-guided/ guided intervention. Participants in the waiting list control group will receive the access data needed to complete the intervention 8 weeks after randomization and will be assigned to the intervention that has shown greater efficiency and user satisfaction. All participants may withdraw from treatment at any time.

Outcome’s assessor, participant and investigator will be blinded. The researcher who administers baseline assessments will be blind to the patients’ treatment group. This researcher will be different from the one who administers the other measures throughout the study.

### Participant timeline an outcomes assessment

Clinical evaluation of program users will be carried out at five times (pre-post and three, six and 12 months after treatment). The instruments covered by the evaluation protocol are aimed at obtaining information necessary to assess whether the change, in addition to having statistical relevance, presents clinical significance and social validation of psychological intervention, allowing to analyze the factors (procedures, goals and results) that will influence the degree of patient satisfaction with respect to the treatment used. Table 1 provides an overview of the measures used at each time point.

**Table 1:** Participant timeline and outcome assessments

Timepoint	Study Period					
	Enrolment	Allocation	Post-allocation			
	-t1	0	t1: Pretest	t2: Posttest	t3: Follow-up 1 (3 months)	t4: Follow-up 2 (6 months)
**Enrolment**						
Eligibility screen	✓					
Informed consent	✓					
Allocation		✓				
**Interventions**						
Transdiagnostic guided internet-delivered intervention with synchronous assistance (UP- guided)						
Transdiagnostic self-guided internet-delivered intervention (UP-unguided)						
Wait list control (WL)						
**Assessments**						
**Primary outcome measure**						
Mini International Neuropsychiatric Interview		✓				
Symptom checklist (SCL-90)	✓			✓		
Beck's Anxiety Inventory (BAI)	✓			✓	✓	✓
Beck's Depression Inventory (BDI-II)	✓			✓	✓	✓
Posttraumatic Stress Disorder Checklist for DSM-5 (PCL-5)	✓			✓	✓	✓
Scale of Difficulties in Emotional Regulation (DERS)	✓			✓	✓	✓
**Secondary outcome measures (during intervention)**						
General Anxiety and the Impairment Severity Scale (OASIS)						
General Depression and the Impairment Gravity Scale (ODSIS)						
**Secondary outcome measures**						
Acceptance/Satisfaction/Usability Measures				✓		

#### Diagnosis interview

*International Neuropsychiatric Interview, Version 5.0.0* (MINI) [43]. It is a structured diagnostic interview. It includes major psychiatric disorders of DSM-IV-R and IDS-10. The Spanish version of Heinze [44] presents a reliability with Kappa de > 0.75.

*Symptom checklist (SCL-90).* Screening instrument to identify symptoms of various psychopathologies. It is made up of 90 items that make up nine dimensions: Somatization, obsessive–compulsive, interpersonal sensitivity, depression, anxiety, hostility, phobic anxiety, paranoia, and psychoticism [45]. Cronbach’s Alpha of all subscales greater than 0.7.

#### Primary outcome measures

*Posttraumatic Stress Disorder Checklist for DSM-5 (PCL-5)* [46]. This instrument describes the symptoms of post-traumatic stress taking into consideration the diagnostic criteria of activation, alterations, avoidance and reexperimentation. It has 20 items that are scored on a Likert-type scale that goes from 0 (not at all) to 4 (totally). In its adaptation to the Mexican population, the psychometric properties of the scale show adequate internal consistency with an alpha of 0.97, as well as an appropriate convergent validity (rs = 0.58 to 0.88) [47]. Items are scored on a Likert scale ranging from 0 to 4, where higher scores indicate more pronounced PTSD symptoms. A cut-off score of 33 was suggested to have a partial diagnosis of PTSD.

*Beck’s Anxiety* Inventory *(BAI)* [48]. Self-administered instrument consisting of 21 questions that determine the severity of symptomatic and behavioral categories of anxious symptomatology present in an individual by means of a four-point scale (0 to 3), where 0 indicates the absence of the symptom, and 3 its maximum severity. These categories correspond to the symptoms that are usually included to make the diagnosis of an anxiety disorder. Previous studies suggest that it is a measure with high internal consistency and construct, divergent and convergent validity [49].

Beck Depression Inventory (BDI-II) [50]. Consists of 21 items that fundamentally evaluate the clinical symptoms of melancholy and the intrusive thoughts present in depression. Among depression measures, it is the one with the highest percentage of cognitive items presented, which is in line with Beck’s cognitive theory of depression. Validated Mexican version of the BDI [51] and for version II (α = 87-0.92) [52].

#### Secondary outcome measures

*Scale of Difficulties in Emotional Regulation* (DERS) [53]. It is a self-applied instrument that measures two dimensions through 15 items, emotional regulation strategies and awareness of emotions. The version validated in Mexican population by De la Rosa et al. [54], presents a Cronbach’s Alpha valued between 0.84—0.74.

*General Anxiety and the Impairment Severity Scale (OASIS)* [55]. It consists of five questions within a scale of 0 to 4, which measures frequency, severity, and avoidance of anxiety in different fields: work / academic interference / family, and deterioration of social and daily life. It has good internal consistency (α = 0.80) and test–retest reliability (k = 0.82). The Spanish language version confirmed the factorial structure and reliability and validity data obtained by the original authors: internal consistency in both populations, in general and clinical (α = 0.86) and test-fail reliability (k = 0.84) [56].

*General Depression and the Impairment Gravity Scale (ODSIS)* [57]. This scale evaluates experiences related to depression. It consists of five items with different answer options ranging from 0 to 4 for each item. It measures the frequency and severity of depression, as well as the level of avoidance to work/academic/home interference, and social life. In the Spanish language version, the internal consistency has proven to be excellent, with a Cronbach alpha between 0.91 and 0.94 and a good convergent and discriminatory validity [56].

#### Opinion measures

*Opinion on Treatment* [58, 59]. This is measured by means of four questions that inform on the level of satisfaction with treatment, (for example “I would recommend treatment to a friend or family member, treatment is considered useful for your case and if you think the treatment was difficult to manage or aversive. On a scale of 1 (nothing) to 10 (very much).

*Usability Scale* (*SUS*) [60]. Measures the usability of a tool, computer program, instrument, etc. It consists of 10 items within a five-point scale, where the 1 is totally at odds and 5 totally agree.

The measures included in the evaluation protocol will be delivered online, except for the mini-Interview, which will be conducted by videoconference or / by telephone by an evaluator.

### Interventions

#### Transdiagnostic guided Internet-delivered intervention with synchronous assistance (UP- guided)

Is an Internet-delivered intervention based on a manualized unified protocol for the transdiagnostic treatment for emotional disorders (to anxiety and mood disorders -unipolar depression-) and derived from stress and trauma disorders structured in a therapist handbook and a patient handbook [61]. The unified protocol incorporates psychological techniques that have proven their effectiveness [14] and includes the following therapeutic modules: 1) motivation for change, understanding emotions, recognition and observation of emotional response; 2) learning to observe experiences, evaluation and re-evaluation of thoughts; 3) what is emotional avoidance, emotion and behavior, and awareness and tolerance of physical sensations; 4) emotional exposure to physical sensations and situations and achievements, maintenance and prevention of relapses.

*E-moción* is a self-applied treatment web system (web/mobile app) based on a transdiagnostic approach for emotional, stress and trauma-derived disorders organized into eight sequential modules (see Table 2), it takes about eight to 12 weeks to complete it. All the modules present the same structure: Module 0. Pre-evaluation, Module 1. Psychoeducation and motivation for change (e.g., understanding emotional reactions to stressors), Module 2. Emotional Coping Skills (p. e.g., emotional regulation), Module 3. Acceptance and awareness-raising skills focused on the present moment (e.g., mindfulness, metaphors); Module 4 and 5 Cognitive coping skills (e.g., cognitive re-evaluation, cognitive flexibility); Module 6 and 7. Behavioral coping skills (e.g., exposure to emotional experiences), Module 8. Achievements, maintenance, and prevention of relapses; Module 9. Post-evaluation. The Internet-delivered treatment program is compatible with desktop devices (PC/Mac), tablet or mobile phone, and will allow participants to access the intervention modules from anywhere and at their own pace. Participants will be encouraged to advance one module per week. The program also sends text messages with motivational content to remind participants to access their modules. Each module includes exercises and tasks for the practice of each technique.

**Table 2:** Components of a transdiagnostic internet-delivered intervention program

Modules	Aims
Module 0	Pre-evaluation
Module 1. Setting goals and staying motivated	Increase motivation and commitment to treatment through the analysis of the benefits and costs of changing and not changing Increase belief in the ability to successfully achieve desired changes (self-efficacy)
Module 2. Understanding emotions	Know the functional nature of emotions and learn about emotional response patterns, including possible factors that maintain them
Module 3. Full emotional awareness	Learn to pay present-focused attention without judging your own experiences
Module 4. Cognitive flexibility	Identify cognitive distortions to subsequently achieve flexibility in the way of thinking, using reinterpretation strategies
Module 5. Opposing emotional behaviors	Identify behaviors that are used to avoid unpleasant emotions and then use alternative actions that approximate those emotions
Module 6. Understanding and coping with physical sensations	Identify physical sensations and develop tolerance to them to reduce the perception of threat
Module 7. Emotional exposures	Increase tolerance to emotions based on exposure to them
Module 8. Recognize your achievements and look to the future	Maintain long-term benefits of treatment and prevent relapse
Module 9	Post-evaluation

#### Synchronous assistance and psychological counseling

To monitor the participant’s progress, each user will be assigned an advisor who will be a health personnel (psychologists) to get a weekly personalized videoconferencing assistance and psychological counseling. The support will be provided by experienced psychologists who will have at least a bachelor’s degree in Clinical Psychology. Before taking part in the trial, they will receive training on the transdiagnostic unified protocol and training in telepsychology to ensure that everyone provides the same support. The role of the psychological advisor is to motivate, guide and listen to the doubts and comments of each participant during one-hour individual online weekly sessions.

#### Transdiagnostic self-guided Internet delivered intervention (UP- unguided)

Self-applied treatment web system based on a transdiagnostic approach for emotional and stress and trauma-derived disorders. The system will contain eight modules (Table 2). The duration of the intervention program may vary between users; however, the participant will have access permits for a maximum period of 12 weeks. To monitor the participant’s progress, at the end of each intervention module, the user will be asked to answer two brief questionnaires to assess anxiety and depression (OASIS, ODSIS). All modules are sequential, allowing the user to go step by step. This arm does not have personalized online assistance.

Under both conditions of intervention, automatic emails with notifications will be sent out to prompt participants to access the intervention if they have not entered within the last 15 days.

#### Waiting list control group (WL)

Participants on the waiting list group will be offered the intervention after two months and will join the Transdiagnostic guided Internet-delivered intervention with synchronous assistance.

## Statistical analysis

### Data management

The requested Personal Data will be processed for research, teaching, and statistical purposes. They will be protected through a code (folio) that guarantees their confidentiality. Access to the data will be limited to the main researcher of the study and the institutional server management technician. Likewise, the data will be kept during the study and once completed, will be kept for an additional period of up to five years to subsequently be eliminated to avoid improper treatment of the same. The information provided through the web and mobile application will be treated with all appropriate security measures, in accordance with the principles contained in the Mexican Federal Law on Protection of Personal Data Held by Private Parties, its Regulations and the Guidelines of the Privacy Notice of the United Mexican States.

### Sample size

For the calculation of the sample size, suggestions reported in the scientific literature regarding the size of the effect in controlled clinical studies where they tested the effectiveness of transdiagnostic interventions for anxiety and depression via online platforms were considered. Cohen’s *d* index will be *used for* this study, as it will be assumed that the variances of the three groups will be homogeneous, otherwise the Hedges *g-index* would be used. In addition, for the calculation of the sample size, since the study will include 3 experimental conditions a One-way ANOVA will be performed for comparison between 3 groups. A conservative approach was adopted and an average magnitude effect size of 0.25 (Cohen’s d) (equivalent to *g* × 0.5), a significance level (α) of 0.05 (*p* < 0.05, 95% confidence) and a conventional statistical power of 80% (1- β 0.8). For a priori analysis, GPower*v3.1.6 software [62] was used and a required sample size of 159 participants (53 per group) was obtained. However, an additional 30% of participants will be recruited keeping in mind attrition rates, as reported in the literature on internet treatments [63, 64]. Thus, the required sample size is estimated to include 207 participants in total (69 participants per group).

### Statistical analysis plan

Descriptive analyses will be carried out to characterize the study sample according to demographic variables: age, sex, occupation, residence, etc. Experimental condition abandonment data associated with diagnosis, intensity of clinical symptoms and sociodemographic characteristics shall be considered. For the analysis of indicators of psychopathology, the intensity of symptomatology, duration of the disorder in months, comorbidity with other psychological problems will be reviewed. To ascertain the homogeneity / heterogeneity in demographic and diagnostic variables that could affect the effectiveness of the study among the two internet-delivered treatment groups (UP-guided vs UP-unguided) and the control group (WL), a statistical analysis will be performed before the intervention. The analysis of the data will be carried out with the SPSS *statistical package in its latest version.* It will be calculated for categorical and continuous variables, the One-way ANOVA. The results shall be presented in three sections: a) contrast analyses to measure the effectiveness of interventions, with specific measures of anxiety, depression, and trauma-stress symptomatology analyzed before and after the online treatment; b) analysis for moderating variable (emotional regulation), and c) acceptance/satisfaction and usability measures of the online intervention. To determine the effectiveness of the intervention program, an analysis of repeated measures variance will be computed through the *SPSS statistical program,* which will compare the pretest measures against posttest measures under the three experimental conditions. The results will be controlled by performing effect size analysis for each intervention group and between treatment groups (UP-guided, UP-unguided) in relation to the control group, through G*Power 3.1.6 software [62]. A conservative approach will be pursued, and the size of the magnitude effect will be estimated using *Cohen’s d index,* a significance level (*α*) of 0.05 (*p* < 0.05, which corresponds to 95% reliability) with a conventional statistical power of 80% (1- β × 0.8). For the analysis of clinical significance associated with clinically significant change, which refers to whether an intervention makes any real difference in people in their daily lives [8], the Reliable Change Rate (ICF) [65] will be calculated, using the criteria of ± 1.64 and ± 1.96 in the area under the normal curve, corresponding to 90 and 95% confidence levels. This method has been chosen because as Jacobson et al. [65] proposes, the amount of statistical change will first be estimated to assess whether the observed difference exceeds the error of measurement of the questionnaire, and second, it will be estimated whether the participant’s score has approached the mean of the functional group. This will be estimated through the normative data of the Mexican version of Beck’s anxiety and depression inventories. Clinical change will be taken as relevant if the participant reduces its levels to < 15 for anxiety, and < 16 for depression. In addition, although the calculation is based on the difference in score means, it incorporates the pre- and post-treatment measurement error. Thus, to assess whether there was a significant clinical change, both criteria must be met: the change must be statistically reliable and clinically relevant. Multiple regression will be used for mediation analyses to test the interaction effects between reference predictor (emotional regulation) and intervention condition, using macro/interface/Process for SPSS.

## Discussion

The present paper describes an Internet-delivered intervention study protocol designed to evaluate the efficacy and acceptability of a transdiagnostic guided Internet-delivered intervention versus a transdiagnostic self-guided Internet-delivered intervention for emotional, trauma and stress-related disorders, and a waiting list in a Mexican adults community sample, and also, to examine the moderators of clinical change, particularly emotional regulation, in the transdiagnostic Internet-delivered intervention for emotional, trauma and stress-related disorders.

Anxiety and mood disorders have been identified as the main causes of psychological disability due to their high prevalence, morbidity and comorbidity in the world and in Mexico; coupled with the impact on the mental health of the population caused by the health contingency due to COVID-19, which has increased the incidence and/or exacerbation of emotional crises and suicidal risk, anxious symptoms, depression and acute stress that could develop if not treated in a timely manner chronically and develop post-traumatic stress, pathological grief and severe and serious emotional disorders. Thus, attention to this priority problem focused on psychological intervention based on a transdiagnostic model that has shown efficacy for the treatment of two or more specific disorders becomes relevant, and thereby contribute to overcoming the inconveniences related to comorbidity between disorders that prevent the full recovery of the person. Likewise, the intervention provided by the Internet will allow better dissemination and will allow addressing the challenge of achieving a greater reach to the vulnerable target population that has not been able to access an effective intervention.

The study has strengths ​​and clinical implications. First, to the best of our knowledge, this is the first Mexican randomized controlled clinical trial to apply a transdiagnostic Internet-delivered intervention to treat EDs and Trauma, stress-related disorders. Second, this study proposes two transdiagnostic Internet-delivered intervention: guided and unguided, to compare the efficacious, acceptance and satisfaction from the users’ view, which will contribute in terms of feasibility of applying the Internet-delivered intervention in different social context to achieve generalization or external validity. Also, the participants will be the direct beneficiaries of the results of the study by reducing anxious and depressive symptoms and will be able to strengthen their coping skills in the face of stressful events.

There are some estimated limitations in the study, one linked to the dropout rate, it is documented [18] that Internet-delivered and self-administered interventions show high dropout rates, particularly participants in the waiting list group. The reasons could be the preference for face-to-face treatments or the lack of access to the Internet or electronic devices to receive the treatment. To reduce this incidence of dropouts, it has been planned to send weekly follow-up reminders and notifications to users to motivate them to continue with the intervention. Another limitation could be the small effect size estimated between the intervention groups because both are based on the same transdiagnostic Internet-delivered intervention and the variant to be compared will be the guide of a clinician who follows the intervention through a weekly videoconferencing session. However, it is estimated that a superiority will be found between the intervention groups compared to the waiting list group.

Finally, the central mechanism of action on the project is the development of a transdiagnostic Internet-delivered intervention program, which can be implemented for dissemination in health centers, universities, community centers, etc. The study is expected to provide evidence of an intervention with content rigorously elaborated and contextualized to society and culture which, with the support of innovative technological resources, provides functional strategies for target users.

### Trial status

The study has not yet started with participant recruitment, it will begin in September 2022. There are no data derived from this study; There are no publications containing the results of this study.

## Data Availability

Not applicable.

## Abbreviations

BAI: Beck Anxiety Inventory
BDI-II: Beck Depression Inventory
CBT: Cognitive Behavioral Treatments.
CONSORT: Consolidated Standards of Reporting Trials
DERS: Scale of Difficulties in Emotional Regulation
DSM-IV-R: Diagnostic and Statistical Manual of Mental Disorders, Fifth Edition, Text Revision
ED: Emotional Disorders.
ICF: Reliable Change Rate
IDS-10: Inventory of Depressive Symptomatology
MINI: International Neuropsychiatric Interview, Version 5.0.0
OASIS: General Anxiety and the Impairment Severity Scale.
ODSIS: General Depression and the Impairment Gravity Scale.
PC: Personal Computer
SPSS: Statistical Package for Social Sciences
SPIRIT: Standard Protocol Items: Recommendations for Intervention Trials
SMP: Mexican Psychologist’s Code of Ethics
SCL-90: Symptom checklist
PCL-5: Posttraumatic Stress Disorder Checklist for DSM-5
PTSD: Posttraumatic Stress Disorder
SUS: Usability Scale
UP: Unified Protocol for the Treatment of Emotional Disorders
